# Interactions among mycorrhizal fungi enhance the early development of a Mediterranean orchid

**DOI:** 10.1007/s00572-023-01118-4

**Published:** 2023-07-12

**Authors:** Jacopo Calevo, Karl J. Duffy

**Affiliations:** Department of Biology, University of Naples Federico II, Complesso Universitario di Monte Sant’Angelo, Via Cinthia, 80126 Naples, Italy

**Keywords:** Fungal growth, In vitro experiments, Mutualism, Priority effect, Rhizoctonias

## Abstract

Orchids depend on mycorrhizal fungi to germinate from seed. While multiple orchid mycorrhizal (OrM) taxa are often found associated with adult orchids, the relative contribution of particular OrM taxa to germination and early orchid development is poorly understood. We isolated 28 OrM fungi associated with the Mediterranean orchid *Anacamptis papilionacea* and tested the efficiency of five isolates on germination and early development, four belonging to the *Tulasnella calospora* species complex and one belonging to *Ceratobasidium*. Co-cultures of varying two-way and three-way combinations of OrM isolates were used in vitro to compare the simultaneous effect on seed germination rate with monocultures. We then tested whether, when given initial priority over other fungi, particular OrM taxa were more effective during the early stages of development. Seedlings germinated with different isolates were transferred to a growth chamber, and either the same or different isolate was added 45 days later. After 3 months, the number of roots, length of the longest root, and tuber area were measured. All OrM fungi resulted in seed germination; however, lower germination rates were associated with the *Ceratobasidium* isolate compared to the tulasnelloid isolates. There was significant decreased germination in co-culture experiments when the *Ceratobasidium* isolate was added. Despite being associated with reduced germination rates, the addition of the *Ceratobasidium* isolate to the seedlings germinated with tulasnelloid strains resulted in significant increased tuber size. Although *A. papilionacea* associates with many OrM taxa, these results show that OrM fungi may play different roles during orchid germination and early development. Even when given initial priority, other fungi may colonize developing orchids and interact to influence early orchid development.

**Supplementary Information:**

The online version contains supplementary material available at 10.1007/s00572-023-01118-4.

## Introduction

Mycorrhizal associations with fungi are estimated to occur in approximately 90% of plants (van der Heijden et al. 2015). Despite their importance for plant life histories, mycorrhizal fungi often go undetected in biodiversity assessments (Soudzilovskaia et al. 2020). This is because the majority of mycorrhizal fungi often do not produce fruiting bodies and are therefore impossible to identify in the field (Egli 2011; Büntgen et al. 2013). Consequently, molecular methods, such as metabarcoding directly from plant root and soil samples, are frequently used to identify the presence of mycorrhizal fungi (Tedersoo et al. 2014, 2020; Waud et al. 2016). While these methods allow us to determine the relative diversity of mycorrhizal fungi associated with a particular plant species or habitat, the contribution of particular mycorrhizal fungi to individual plant fitness and their interactions with other mycorrhizal fungi remains poorly understood (Bidartondo et al. 2004; McCormick et al. 2009). Hence, in order to understand the function of particular mycorrhizal taxa and how they interact to influence plant fitness, it is necessary to isolate fungi and study their effect on plant growth (Selosse et al. 2018).

Orchids are renowned for their obligate dependence on particular mycorrhizal fungi, orchid mycorrhizal (OrM) fungi (Selosse et al. 2011, 2017). This is because their seeds almost completely lack endosperm, so in order to germinate, they need OrM fungi to transfer nutrients and water during early growth phases (Arditti and Ghani 2000). They often also require OrM for subsequent development to adult plants. Most adult orchids retain their association with OrM fungi throughout their life (Rasmussen and Rasmussen 2009; Swarts and Dixon 2009; Dearnaley et al. 2012). Despite this dependence on OrM fungi, orchid species may differ greatly in their level of reliance on OrM fungi and on which OrM taxa they associate with (Waud et al. 2017; Duffy et al. 2019). Some orchids remain associated with a single fungal symbiont (Swarts et al. 2010; Davis et al. 2015), while others undergo a facultative or obligatory turnover of OrM taxa during the adult stage or in response to environmental disturbance (McCormick et al. 2016; Ventre Lespiaucq et al. 2021). Some orchids even associate with a greater diversity of OrM fungi in adulthood (Bidartondo and Read 2008; Rasmussen et al. 2015), thereby obscuring the nature of their ecological dependency on particular fungi. Even orchid species that mainly associate with one or few fungal taxa can be colonized by other OrM fungi during their life cycle (Calevo et al. 2020, 2021a). This is because OrM fungi may occupy different ecological niches with access to a wider breadth of soil nutrients (Nurfadilah et al. 2013; Mujica et al. 2016; Mujica et al. 2020; Vogt‐Schilb et al. 2020; Kaur et al. 2021). Hence, mycorrhizal assemblages may provide better access to scarce nutrients, potentially increasing germination success and development into the adult stage. However, understanding the influence of particular OrM fungal taxa at different life history stages is critical for effective orchid conservation (Fay 2018) as we will be able to better predict what OrM fungi are important for the establishment of rare orchids (Reiter et al. 2018).

In vitro experiments are commonly used to test the efficacy of particular OrM fungi on seed germination rates. Most studies using symbiotic seed germination have applied single fungal cultures (i.e., single OrM taxa) isolated from adult roots (Rasmussen 2002; Khamchatra et al. 2016; Calevo et al. 2020). These studies have demonstrated that OrM fungi isolated from adult roots promote germination but do not necessarily support subsequent seedling growth (Rasmussen et al. 2015), with some OrM taxa even associated with increased protocorm mortality (Rasmussen 2002; Adamo et al. 2020). Fungal inocula that comprise various OrM taxa (co-cultures), as opposed to those comprised of single taxa (monocultures), in theory, should positively influence seed germination and development compared to monocultures. This is because they contain a diverse sample of the OrM community and may be more likely to include the most advantageous OrM partner to increase germination success. While biotic interactions, such as competition among OrM taxa, may influence orchid germination success (Shao et al. 2020a), it could be that, due to chance, some fungi have initial priority to colonize orchid seeds, and orchid germination rate may be affected as a result of a priority effect among OrM taxa (Peay et al. 2012). However, little is known about the interactions among OrM fungi during the germination and early development of orchids or whether priority effects are common, with only a few studies using fungal co-cultures simultaneously to test for their influence on orchid seed germination (Sharma et al. 2003; Wang et al. 2016; Shao et al. 2020a).

**Fig. 1:**
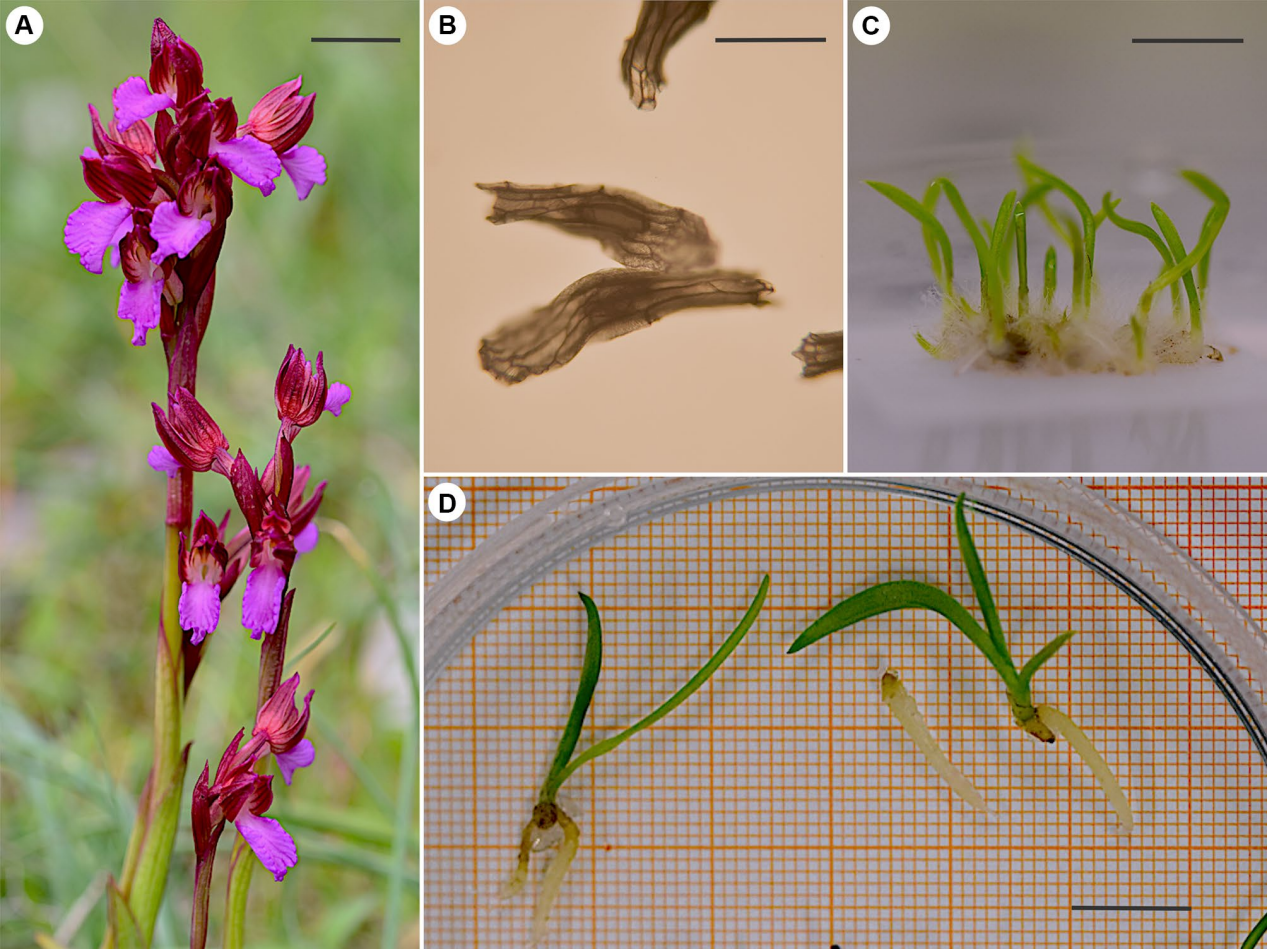
The **a** habit of *A. papilionacea*, scale = 2 cm; **b** embryo-containing seed, scale = 200 µm; **c** seedlings with first leaves on symbiotic media, scale = 1 cm; and **d** seedlings after the experiment in the growth chamber, scale = 1 cm

Here, we investigate the efficacy of different mycorrhizal fungal taxa associated with the Mediterranean orchid, *Anacamptis papilionacea* (L.) R.M.Bateman, Pridgeon and M.W.Chase, in germination and early growth. *Anacamptis papilionacea*, a species listed as Least Concern in the European IUCN Red List (Rankou 2011), flowers between April and May in Italy and produces an inflorescence of up to 20 purple, nectarless flowers (Fig. 1a). Previous work describing the OrM communities associated with *A. papilionacea* using metabarcoding suggested that it associates with OrM mainly belonging to the Ceratobasidiaceae and Tulasnellaceae (Jacquemyn et al. 2014). We isolated different OrM taxa from the roots of adult *A. papilionacea* and performed manipulative experiments with mono- and co-cultures of OrM taxa to determine how different OrM taxa influence its seed germination and seedling development. Specifically, we tested whether (i) monocultures of different OrM fungi isolated from adult roots initiated orchid seed germination equally, (ii) co-cultures of OrM isolates result in increased germination rates compared to monocultures, and (iii) the order in which fungi colonize the orchid during its post-germination development (i.e., the priority effect) influences early growth as measured by the number of roots produced, length of the primary root, and tuber area.

## Materials and methods

### Seed collection and fungal isolation

In May 2020, we collected seeds of *A. papilionacea* (Fig. 1b) from 18 plants from a population on Mount Vesuvius near Naples in Southern Italy and five plants from one population in Northern Italy at Monte Santa Croce, near Genoa. Seeds were kept in paper envelopes to dry and kept at 4 °C until use. For fungal isolation and comparison, four roots were collected from the Mount Vesuvius population (in April 2021), and seven roots (ranging from 2 to 11 cm) were collected from the Monte Santa Croce population (in April 2020). The Monte Santa Croce population is a small population in a *Festuco-Brometalia* habitat type surrounded by introduced *Pinus nigra*, on clay calcareous soil type; the Vesuvius population is larger and grows in sympatry with other orchids such as *A. morio*, being surrounded by small shrubs (such as *Ginestra* sp.) on volcanic bedrock covered with mosses and lichens.

Roots were rinsed under tap water to remove soil and debris, and the surface was sterilized in a 1% sodium hypochlorite solution for 3 min, followed by three washes in sterile dH_2_O for 10 min each (Swarts and Dixon 2017). Roots were hand sectioned under a sterile hood, and each section was cut into two or four fragments, depending on the diameter of the root. A total number of 692 root fragments were plated in Petri dishes containing a half concentration of potato dextrose broth (Condalab, Madrid) with 10 g/l of agar (Potato Dextrose Agar, PDA) and 5 ml/l streptomycin (1 g of streptomycin dissolved in 50 ml of sterile dH_2_O and filtered) to inhibit bacterial growth on plates. As soon as fungal hyphae emerged, subcultures of each fungus were taken to obtain pure fungal isolates. Fungal isolates that, despite the use of antibiotics and subcultures, had bacterial contamination were subjected to the cabin-sequestering method (Shi et al. 2019). For this, 5-mm square holes (the “cabin”) were cut on a solid 1/2 PDA plate enriched with streptomycin, and a 3 × 3-mm PDA plug containing the contaminated fungal isolate was inoculated into the cabin; the cabin was then covered with a sterilized 22 × 22-mm microscope glass cover, and the Petri dish was incubated at 20 ± 2 °C. When the hyphae were growing out along the edge of the coverslip, they were collected for subcultures. A total of 84 isolates were obtained from the root samples. After morphological screening to assess whether they were presenting morphology similar to OrM endophytes (rhizoctonias), 28 isolates from Monte Santa Croce and five from Mount Vesuvius were stored at 4 °C, and their mycelia were extracted for molecular identification.

### DNA extraction and molecular identification of isolates

Mycelia of the fungal isolates were grown in flasks with half-concentration potato dextrose broth (PDB) liquid medium for 3 to 7 days (depending on fungal growth) and collected in 2-ml tubes for DNA extraction. Total DNA was extracted with the NucleoSpin Plant II kit (MACHEREY–NAGEL, Düren, Germany) according to the manufacturer’s instructions. The quality and quantity of DNA samples were assessed by spectrophotometry (ND-1000 Spectrophotometer NanoDropH; Thermo Scientific, Germany). The nuclear ribosomal ITS region was amplified from all DNA extracts with the ITS1-ITS4 fungal primers (White et al. 1990). The ITS representative sequences of OM isolates generated in this study were submitted to GenBank and recorded under the following string of accession numbers: OM971071-OM971074 for Ceratobasidiaceae and OM976746-OM976769 for Tulasnellaceae.

### Phylogenetic analyses of isolates

Maximum likelihood (ML) analyses were carried out with the representative sequences of the tulasnelloid and ceratobasidioid OTUs. Sequences included in the ML analyses comprised the best BLAST hits as well as fungal sequences from a variety of terrestrial orchids, including the target species, from different continents and environments, as well as from non-orchid plants, fungal mycelia, and fruiting bodies. Sequences were aligned using the program MAFFT v.7.490 (Katoh and Toh 2010) and then trimmed with trimAl v.1.2.59 (Capella-Gutiérrez et al. 2009) using automated selection on “gappyout: mode. ML estimation was performed with RAxML v. 8.2.12 (Stamatakis 2014) through 1000 bootstrap replicates (Felsenstein 1985) using the GTR + GAMMA algorithm to perform a tree inference and search for a good topology. Sequence alignment, trimming, and ML estimation were performed on CIPRES Science Gateway (Miller et al. 2011). Support values from bootstrapping runs were mapped on the globally best tree using the − *f* option of RAxML and −  × 12,345 as a random seed. Nodes receiving a bootstrap support < 70% were not considered as well supported.

### Seed germination with mono- and co-cultures

**Fig. 2:**
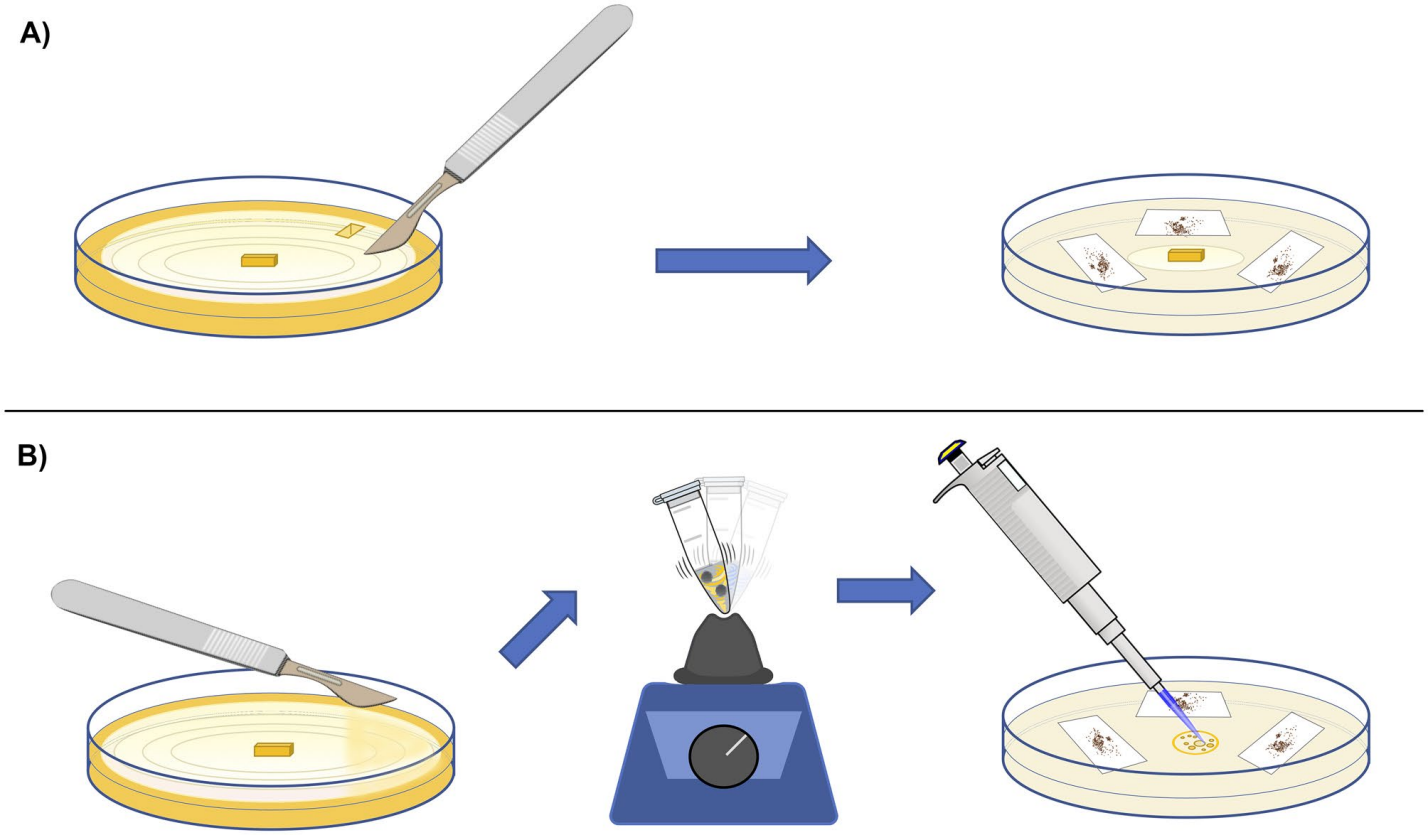
Symbiotic seed sowing performed by using **A** the classical fungal monoculture plug as described by Ercole et al. (2015b) and **B** the described co-culture liquid technique where the mycelia were collected and inserted in a 2-ml sterile tube then disrupted by vortexing with sterile beads prior to plating onto OA medium with a pipette

Seeds were sterilized using a 1% sodium hypochlorite solution for 20 min and then rinsed three times in sterile dH_2_O for 10 min each. Seeds were then sowed in four Petri dishes with sterile oat agar (OA) medium with the mono-fungal inoculum as described by Ercole et al. (2015b) (Fig. 2a). This was repeated for each isolate as preliminary analysis (by using *A. papilionacea* seeds from Monte Santa Croce), and five random isolates showing a difference in germination performance were selected for the experiment for which seeds from the Vesuvius population were used, given the quantity needed.

To test whether interactions among isolates influence seed germination rates, we prepared inocula with different combinations of the five isolates for symbiotic seed germination. We prepared inocula containing combinations of two and three isolates separately and in all possible isolate combinations. For this, the fungal plug was substituted with a drop of a 1% sterile agar solution in which the mycelium was collected and disrupted with sterile beads by vortexing (Fig. 2b). A negative control, in which no fungal inoculum was added to OA, and an asymbiotic control, by using the BM-1 orchid medium, were used. Each sowing treatment was replicated three times. Approximately 150 seeds were placed over the three layers of filter paper of each Petri dish. The exact number of seeds in each Petri dish was counted by acquiring images with a stereomicroscope Leica MZ6 camera and automatically analyzing them with the ImageJ software (Schneider et al. 2012). Images that contained overlapping seeds were manually counted. Random images were also manually checked to verify accuracy. A total of approximately 36,000 seeds (mean = 127.5 ± 40.7 SD per quadrant) were counted. In addition to seed sowing, after each sterilization, a subset of approximately 100 seeds was used to test their viability using tri-phenyl tetrazolium chloride (TTC) (Custódio et al. 2016).

To test whether isolates differed in their capacity to initiate seed germination, we calculated the proportion of seeds germinated as the number of seeds that reached growth stage four, where the seedlings produce the first leaves, from the number of seeds sowed on each plate (Fig. 1c). We tested separately whether the proportion of viable seed germinated varied according to (i) monocultures of OrM isolates and inocula containing co-cultures of (ii) all combinations of two isolates together and (iii) all combinations of three isolates together. The first analysis addresses the hypothesis that OrM fungi differ in their capacity to initiate seed germination. As negative controls resulted in no seed germination, we excluded these from formal analyses. The second two experiments address the hypothesis that there are interactions among OrM fungi which can result in differences in germination success. As seed germination data were recorded as an “events out of trials” structure (i.e., the number of viable seeds produced from the total number of seeds), we used generalized linear models (GLMs) using the “glm” function in R (R Core Team 2021) with a quasibinomial error distribution to account for overdispersion and a logit link. To assess whether response variables varied according to OrM isolates overall, we used the “anova” function in the car package (Fox and Weisberg 2018) to obtain a type III sum of squares output for the GLM model. Differences between treatment means were compared using post hoc tests using the package “emmeans” (Lenth 2020). Family-wise error rate was controlled using the Tukey HSD corrections.

### Seedling development and timing of OrM colonization

Forty-five days after sowing, Petri dishes containing symbiotically germinated seeds with individual isolates were transferred to a controlled plant growth chamber (Senova CMC-B450, Senova Biotech, Shanghai) and subjected to the following light and temperature cycles: 15 min at 2400 lx, 17.5 °C and 60% relative humidity (RH); 2 h and 45 min at 5000 lx, 18 °C and 58% RH; 5 h at 10,000 lx, 20 °C and 53% RH; 5 h at 15,000 lx, 22 °C and 50% rh; 1 h at 10,000 lx, 20 °C and 52% RH; 1 h at 5000 lx, 20 °C and 53% RH; 1 h at 2400 lx, 18 °C and 55% RH; and a final step of 9 h at 0 lx, 17.5 °C and 60% RH. Approximately 2 weeks later, 2 months after initial sowing, developed seedling percentage of each Petri dish was calculated as the average of the three quadrants, while the total germination of each species was calculated as the average of germination percentage of individual Petri dishes ± SE (standard error) corrected per seed viability.

To test whether fungi differ in their capacity to initiate early orchid growth, 45 seedlings (Fig. 1c) grown with separate monocultures of each OrM isolate were then transferred to plastic jars with OA medium and re-inoculated with the same original fungus or each of the other isolates, with nine replicates for each treatment. Jars were then subjected to the same cycle conditions in the growth chamber, and seedlings were collected after 3 months for growth data collection (Fig. 1d). We used three measures to measure orchid growth when the first fungus was added to the seedlings: (i) the proportion of roots produced, as there is a limit on the number of roots that can be produced, we used the maximum of four roots that were produced, (ii) the length of longest root (mm), and (iii) the tuber area (mm^2^). Measurements of orchid roots and tuber area were made by analyzing photos with ImageJ (Schneider et al. 2012). The number of roots produced was analyzed with a GLM with a Poisson error structure and a log link. The length of the longest root and tuber area (log10 transformed) were analyzed using two-way type III ANOVA in the “car” package in R. We tested the interaction between the initial fungus added at germination (T_0_) and the fungus added during development (T_1_). We removed the interaction term if it was not significant. Differences between treatment means were compared using post hoc tests in the package “emmeans.” Family-wise error rate was controlled using the Tukey HSD corrections.

## Results

### Isolated OrM fungi

Sequences obtained from 28 isolates from the Monte Santa Croce population resulted in two groups, belonging to tulasnelloid and ceratobasidioid fungi. All tulasnelloid fungi clustered in the *Tulasnella calospora* species complex (Supplementary Fig. 1a). The ceratobasidioid fungi were found to be members of *Ceratobasidium* and closely related to fungi previously retrieved from another orchid species *Anacamptis morio* (Ercole et al. 2015a) with which it showed a 99.52% sequence similarity (Supplementary Fig. 1b). Among these, five were selected for the experiment as representative of clades with 98% sequence identity. For simplicity, we hereafter refer to these fungi as cer2.20 for the *Ceratobasidium* strain and tul2.13, tul2.28, tul7.1, and tul7.2 for the four *T. calospora* isolates.

Five additional isolates were obtained from Vesuvius, none of which belonged to Tulasnellaceae but to Ceratobasidiaceae, with one isolate clustering with the above-mentioned *Ceratobasidium* sp. found in samples from Monte Santa Croce and the other clustering separately (Supplementary Fig. 1b).

### Seed germination with mono- and co-cultures

**Fig. 3:**
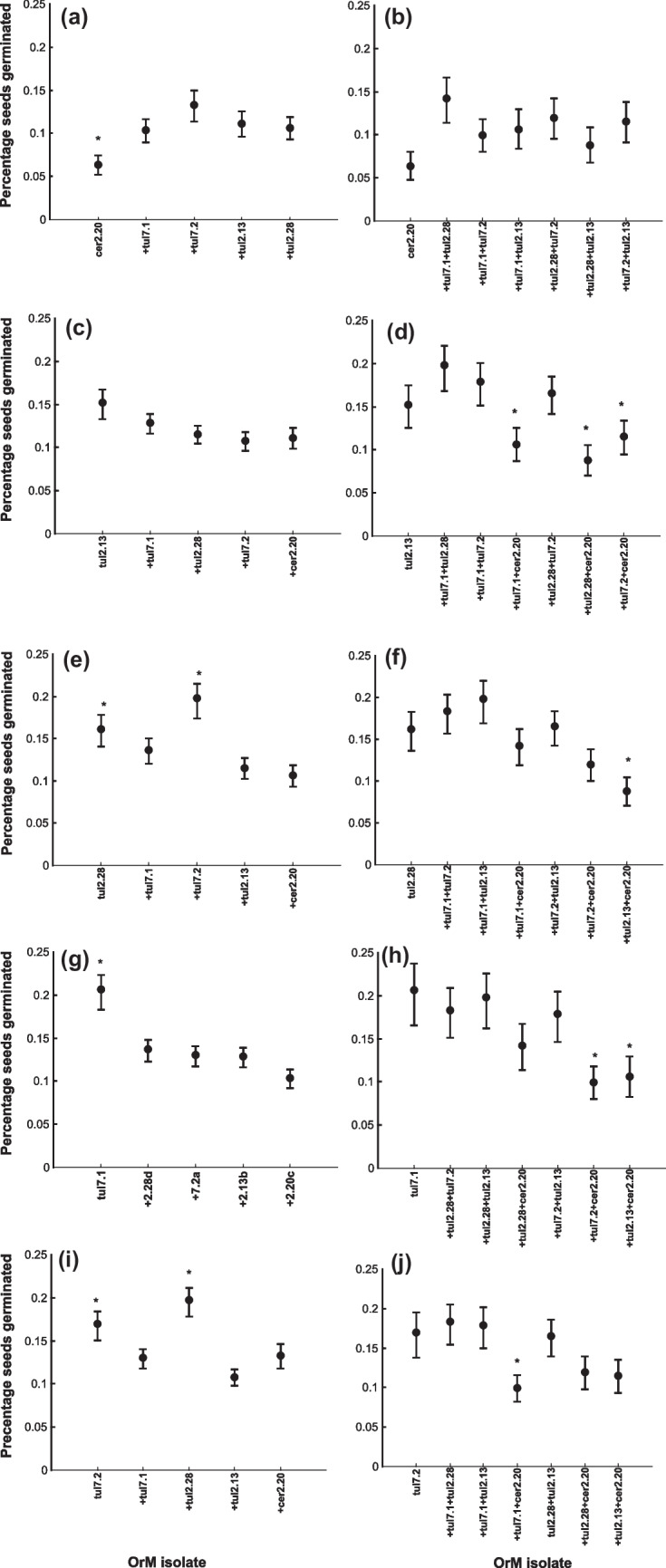
Proportion of seeds germinated in different OrM mono- and co-cultures; **a** cer2.20 and two-isolate co-cultures, **b** cer2.20 and three-isolate co-cultures, **c** tul2.13 and two-isolate co-cultures, **d** tul2.13 and three-isolate co-cultures, **e** tul2.28 and two-isolate co-cultures, **f** tul2.28 and three-isolate co-cultures, **g** tul7.1 and two-isolate co-cultures, **h** tul7.1 and three-isolate co-cultures, **i** tul7.2 and two-isolate co-cultures, **j** tul7.2 and three-isolate co-cultures. Asterisks denote the significant effect of culture on germination rates. **a**, **d**, **f**, **h**, **j** The negative effect of cer2.20 on the germination rates either in mono- and co-cultures is statistically significant (*p* < 0.01). In **g** and **i** the positive effect of *T. calospora* isolates on germinations is statistically significant (*p* < 0.01)

All OrM isolates initiated seed germination. Seed viability tests using TTC showed that *A. papilionacea* had a mean seed viability of 64.2% (± 4.5%). There was no seed germination in any negative control, indicating that seeds of *A. papilionacea* depend on OrM fungi to germinate. The proportion of germinated seeds that reached the photosynthetic stage varied according to OrM isolate. Overall, only a small proportion of viable seeds reached the photosynthetic stage (Fig. 3), indicating that many seeds were not able to germinate or develop after initial germination. The ceratobasidioid isolate cer2.20 resulted in fewer germinated seeds compared with seeds germinated with OrM isolates from the *T. calospora* complex (overall *χ*^2^ = 109.8, df = 4; *p* < 0.001) and tul7.1 resulting in higher germination than tul2.13 (post hoc test, *p* = 0.019).

Comparison of two-isolate co-cultures with cer2.20 monoculture indicated that there were differences in germination rates compared with co-cultures containing one of the *T. calospora* isolates (*χ*^2^ = 14.79, df = 4; *p* = 0.005; Fig. 3a). Post hoc tests showed that there were significantly higher germination rates when cer2.20 was added with tul7.2 (*p* = 0.003; Fig. 3a) and tul2.13 (*p* = 0.045; Fig. 3a). However, there was no difference in germination rates when combined in three-isolate co-cultures with any combination of other isolates (*χ*^2^ = 9.99, df = 6; *p* = 0.125; Fig. 3b).

Monocultures of tul2.13 did not differ in germination rate compared with two-isolate co-cultures (*χ*^2^ = 8.32, df = 4; *p* = 0.08; Fig. 3c), while three-isolate co-cultures containing tul2.13 varied significantly (*χ*^2^ = 27.38; df = 6; *p* < 0.001; Fig. 3d). Post hoc tests showed that there was increased germination when tul2.13 was combined with tul7.2 + tul2.28 compared with co-cultures containing tul7.1 + cer2.20 (*p* = 0.019; Fig. 3d), tul2.28 + cer2.20 (*p* = 0.001; Fig. 3d), and tul7.2 + cer.220 (*p* = 0.047; Fig. 3d). Likewise, co-cultures containing tul2.13 combined with tul7.1 + tul7.2 resulted in increased germination compared with co-cultures containing tul2.13 combined with tul228 + cer2.20 (*p* = 0.008; Fig. 3d).

Seed germination rates with monocultures of tul2.28 varied compared with two-isolate co-cultures (*χ*^2^ = 27.61, df = 4; *p* < 0.001; Fig. 3e). Post hoc tests revealed that there was increased germination when combined with tul7.2 compared with when combined with tul7.1 (*p* = 0.036; Fig. 3e), tul2.13 (*p* = 0.001; Fig. 3e), and cer2.20 (*p* < 0.001; Fig. 3e). In addition, the monoculture containing tul2.28 resulted in increased germination than compared with the co-culture containing cer2.20 (*p* = 0.042; Fig. 3e). Similarly, seed germination rates varied according to three-isolate co-cultures (*χ*^2^ = 25.31, df = 6; *p* < 0.001; Fig. 3f). Post hoc tests showed that co-cultures containing tul2.28 combined with tul2.13 + cer2.20 resulted in lower germination rates than those containing tul2.28 combined with tul7.1 + tul7.2, tul7.1 + tul2.13, and tul7.2 + tul2.13 (*p* < 0.01 for all comparisons; Fig. 3f).

Seed germination rates with monocultures of tul7.1 varied compared with two-isolate co-cultures (*χ*^2^ = 33.32, df = 4; *p* < 0.001; Fig. 3g), with tul7.1 resulting in higher seed germination compared to co-cultures containing other isolates (*p* < 0.01 for all comparisons; Fig. 3g). Seed germination rates also varied with monocultures of tul7.1 compared with three-isolate co-cultures (*χ*^2^ = 21.51, df = 6; *p* = 0.001; Fig. 3h). There was decreased germination when combined with tul7.2 and cer2.20 (*p* = 0.029) and tul2.13 and cer2.20 (p = 0.027) (Fig. 3h). 

Likewise, seed germination rates varied with monocultures of tul7.2 and co-cultures when combined with another isolate (*χ*^2^ = 37.76, df = 4; *p* < 0.001; Fig. 3i). There were increased germination rates when combined with tul2.28 compared with when combined with tul7.1 (*p* = 0.001; Fig. 3i), tul2.13 (*p* < 0.001), and cer2.20 (*p* = 0.004). However, the tul7.2 monoculture resulted in increased germination compared with the co-culture containing tul2.13 (*p* = 0.003). Seed germination rates varied according to three-isolate co-cultures and tul7.2 (*χ*^2^ = 19.36, df = 6; *p* < 0.003; Fig. 3j) with co-cultures containing tul7.2 + tul7.1 + cer2.20 that produced fewer germinated seeds than co-cultures combined with tul7.2 + tul7.1 + tul2.28 (*p* = 0.025; Fig. 3j) and tul7.2 + tul7.1 + tul2.13 (*p* = 0.045; Fig. 3j).

### Seedling development and timing of OrM colonization

**Fig. 4:**
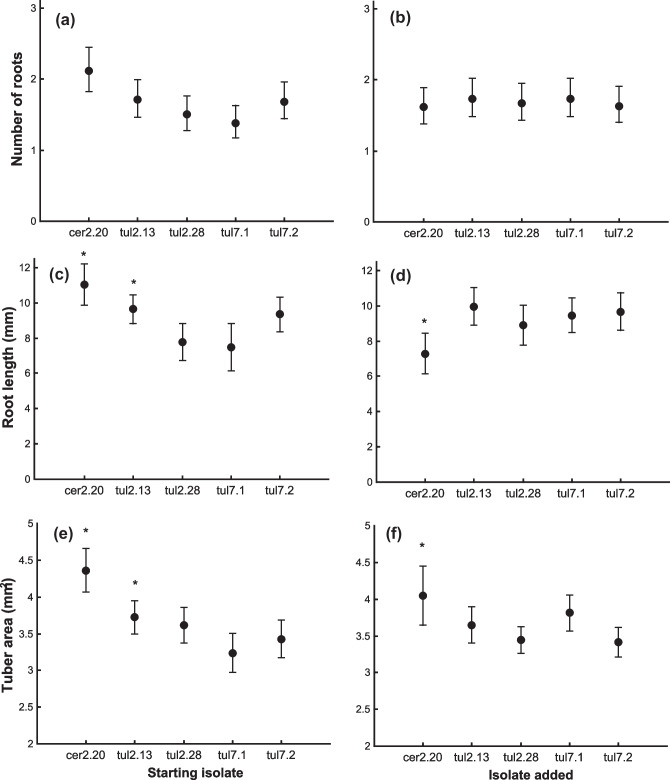
Relationships between starting OrM isolates (**a**, **c**, **e**) and OrM isolates added 45 days later (**b**, **d**, **f**) and **a** proportion of roots produced, **b** length of the primary root (mm), and **c** the area of the tuber (mm.^2^). Asterisks signify isolates that had a significant positive effect on growth rates (**c**, **f**), while the asterisk signifies the negative effect of cer2.20 on root length (**d**)

There was no interaction between starting isolate and the isolate added after 45 days on any response variable; therefore, we removed the interaction term from our models. The proportion of roots produced did not vary according to starting isolate (*χ*^2^ = 8.12; df = 4; *p* = 0.087: Fig. 4a) or by the addition of an isolate (*χ*^2^ = 33; df = 4; *p* = 0.987). The length of the primary root was positively influenced by the starting isolate cer2.20 compared to tul7.1 and tul2.28 (*p* < 0.001; Fig. 4b), while the tul2.13 isolate resulted in increased root length compared to tul7.1 (*p* = 0.023; Fig. 4b). However, when added during the experiment, cer2.20 reduced root length compared with tul2.13, tul2.28, and tul7.1 (*p* < 0.01: Fig. 4b), indicating that adding cer2.20 may impede root lengthening during development. Tuber area was positively associated with cer2.20 as a starting isolate compared with all other isolates (*p* < 0.01: Fig. 4c), while tul2.13 resulted in increased tuber area compared with tul7.1 (Fig. 4c). When added during the experiment, cer2.20 resulted in increased tuber growth compared with the isolates tul2.28 and tul7.2 (*p* < 0.03; Fig. 4c).

## Discussion

Isolation of mycorrhizal fungi from adult roots of *A. papilionacea*, coupled with symbiotic seed germination experiments with five isolates, has revealed complex interactions between OrM fungi and their effect on germination rates and early orchid development. While *A. papilionacea* associates both with tulasnelloid and ceratobasidioid fungi, it probably depends on both OrM groups for successful establishment in natural populations. Indeed, Jacquemyn et al. (2014) found that members of the Ceratobasidiaceae and Tulasnellaceae were mainly associated with *A. papilionacea* in different populations in Southern Italy. As several fungal taxa are often found on orchid protocorms and seedlings simultaneously (Bidartondo and Read 2008), it could be that multiple mycorrhizal taxa function together to promote seed germination and subsequent seedling recruitment. However, Shao et al. (2020b) found that OrM co-cultures did not result in higher seed germination rates and seedling development of *Dendrobium nobile* compared to monocultures. Likewise, Gao et al. (2020) found that one *Ceratobasidium* taxon was responsible for germination to stage four (i.e., photosynthetic plant), despite other OrM taxa being able to initiate seed germination to stage two. In *A. papilionacea*, there was a clear difference between ceratobasidioid fungus and tulasnelloind fungi in seed germination rates, with the *Ceratobasidium* isolate less efficient in germinating seeds compared with different isolates of *T. calospora*, particularly the isolate tul7.1. Hence, co-cultures of OrM are not necessarily more efficient at germinating orchid seeds compared with monocultures. These results suggest that the quality (i.e., the taxonomic composition) of OrM assemblages, rather than their quantity, may be an important factor in seed germination.

Differences in the effectiveness of OrM taxa were found also in our growth experiment. Even though tulasnelloid fungi resulted in higher germination rates, they were less efficient in developing roots and tubers compared to the ceratobasidioid fungus. This reduction in symbiont efficiency after germination has been previously described in seed germination experiments with Tulasnellaceae taxa (i.e., Yamamoto et al. 2017; Meng et al. 2019). It was assumed in these studies that the lack of further development was caused by an inadequate host-fungus compatibility, since the OrM fungi used were isolated either from a different orchid species or from adult plants. However, Fuji et al. (2020) suggested that symbiotic compatibility between orchids and OrM taxa should not necessarily be determined solely based on seed germination percentages. Indeed, the importance of Tulasnellaceae in our study is evident both by their efficiency in germination and by the number of isolates obtained from adult plants. On the other hand, we found that the cer2.20 taxon resulted in lower germination rates of seeds but increased seedling development and was associated with an overall increase in tuber size.

Despite the efficiency of cer2.20 in seedling development in terms of the number of roots produced and tuber size, its addition at T_1_ significantly reduced the length of the primary root. In developing plants, the root is the primary organ for acquiring nutrients and water from soil; thus, the architecture and length of the root system play a crucial role in survival under conditions of nutrient deficiency or drought (Koevoets et al. 2016; Sun et al. 2020). Recently, Han et al. (2018) evaluated the effects of glycine as an exogenous source of nitrogen on root growth and NO_3_^−^–N uptake in *Brassica campestris*. They found that the addition of glycine decreased seedling root length and reduced ^15^NO_3_^−^–N uptake but increased the number of root tips per unit root length. Orchids require an exogenous source of carbon and nitrogen which is supplied through symbiosis with OrM fungi. Fochi et al. (2017) suggested that organic N may be the main form of nitrogen transferred to the orchid host by OrM fungi in the form of amino acids as indicated by the upregulation of plant amino acid transporters, as well as enzymes involved in amino acid breakdown in developing orchids. Hence, the reduced length in primary root elongation associated with the presence of cer2.20 may be because they already have a more efficient nutrient uptake compared to seedlings germinated with tulasnelloid strains owing to their higher number of shorter roots and larger tuber size.

The order in which OrM strains colonize the orchid may influence subsequent growth rates. Here, we detected differences in the effect that these strains have on both germination and subsequent growth of *A. papilionacea*. Hence, while a priority effect may occur in OrM fungi and influence orchid growth, the interactions among different strains of fungi are probably more important for orchid development. Ceratobasidiaceae are both functionally and taxonomically highly diverse, and all major nutritional modes are conserved in the family. Orchid root symbionts phylogenetically overlap with putative saprotrophs from soil samples, suggesting that saprotrophic strains from natural soils are more easily accessible for orchids (Veldre et al. 2013). On the other hand, some *Tulasnella* taxa have been considered soil saprotrophs, although this suggestion has been debated (Calevo et al. 2020, 2021b; Egidi et al. 2018; Voyron et al. 2017), particularly within the *Tulasnella calospora* species complex (Egidi et al. 2018; Voyron et al. 2017). Even where OrM fungi are present, failure to reach adulthood could be due to the presence of less effective OrM taxa that are not able to support complete seed germination to the photosynthetic stage (Batty et al. 2001). It could be that some members of the Tulasnellaceae are less competitive in soil and might be harbored by adult plants, therefore being more accessible for orchid seeds (Calevo et al. 2021b). Seeds or protocorms that encounter the suitable *Ceratobasidium* taxa can then develop tubers rapidly, thus having more chances to be recruited.

These results shed light on the interactions between the early stages of orchid growth and OrM fungi showing that, at least in the generalist *A. papilionacea*, germination rate and seedling development are affected by a priority effect among OrM fungi, with seeds colonized by tulasnelloid fungi having more chances to germinate and those by the ceratobasidioid fungus to develop quicker. The long-term conservation of both the orchid and fungal diversity is an important issue, with the success of re-introductions of orchids into former habitats dependent on the presence and abundance of suitable mycorrhizal fungi (Vogt‐Schilb et al. 2020). *Tulasnella calospora* and members of *Ceratobasidium* are likely to have wide distributions, wider than the distribution of *A. papilionacea* (Jacquemyn et al. 2017; Waud et al. 2017), although fungal distribution is still poorly understood due to taxonomic uncertainties.

As more than one OrM taxon can induce germination of *A. papilionacea* seeds in vitro, future studies should focus on investigating OrM efficiency in semi-natural and natural conditions and test if the introduction of multiple fungal inoculants could be beneficial in either translocation or restoration experiments to reduce orchid recruitment losses.

In conclusion, these results show that *A. papilionacea* associates with OrM taxa from different fungal families, and these taxa are able to initiate germination and early growth. However, OrM taxa differ in their efficiency during different stages of orchid development, and their order of arrival during early orchid growth may influence recruitment success.

## Supplementary Information

Below is the link to the electronic supplementary material.

Supplementary file1 (DOCX 205 KB)

## Data Availability

ITS sequences of the fungal isolates used in this study are deposited in GenBank under the accession numbers OM971071-OM971074 for Ceratobasidiaceae and OM976746-OM976769 for Tulasnellaceae.
