# Supplementary material for: Interactions among mycorrhizal fungi enhance the early development of a Mediterranean orchid
Source: Mycorrhiza. 2023 Jul 12;33(4):229–40. doi: 10.1007/s00572-023-01118-4 (PMC10442268; doi:10.1007/s00572-023-01118-4)
Supplement: Supplementary file 1 — Supplementary file1 (DOCX 205 KB) [file 572_2023_1118_MOESM1_ESM.docx]

**Supplementary Information**

**Title: Interactions among mycorrhizal fungi enhance the early development of a Mediterranean orchid**

Authors: Jacopo Calevo and Karl J. Duffy

Corresponding author: jacopo.calevo@gmail.com

Journal: *Mycorrhiza*


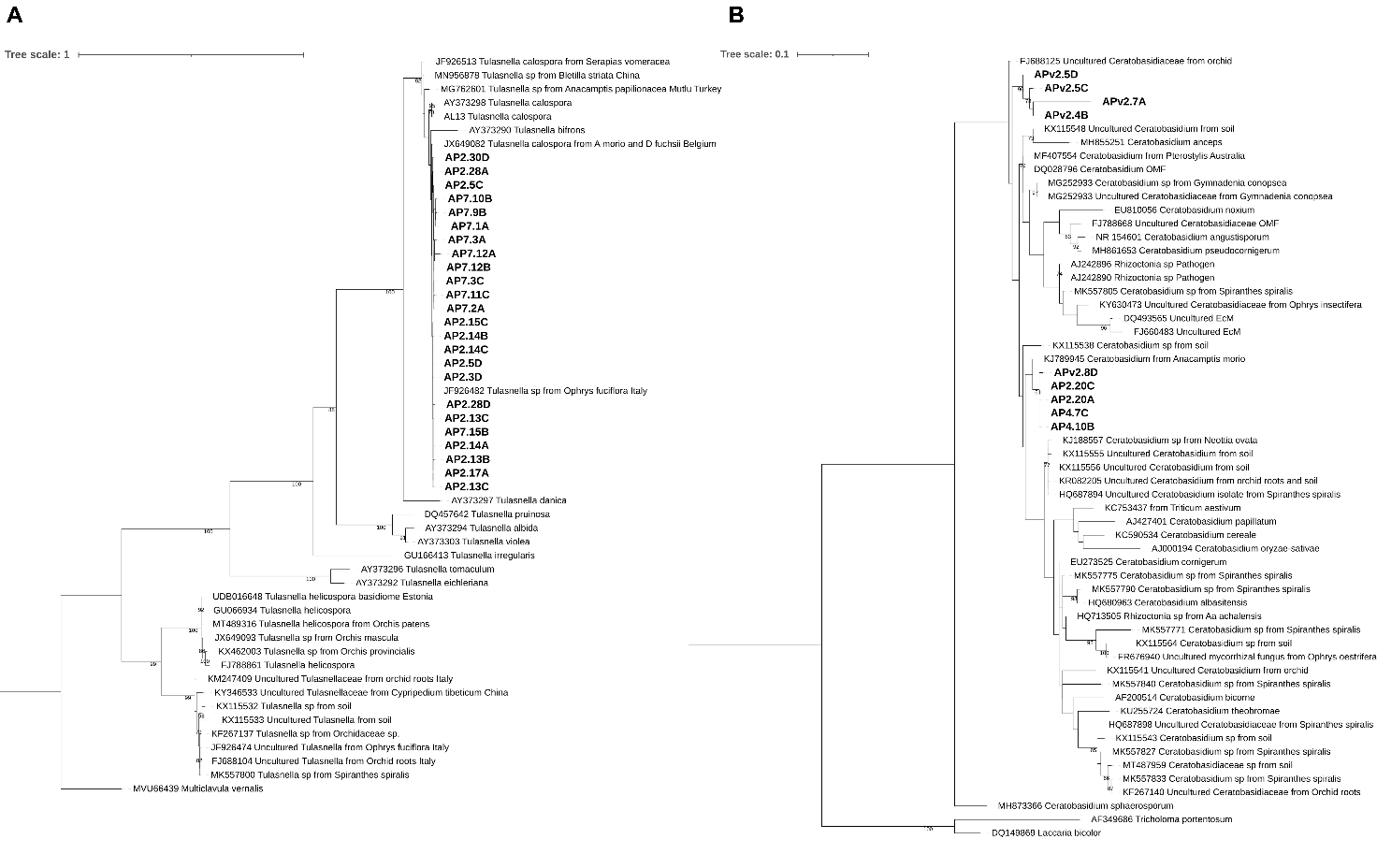


**Supplementary Fig. 1** Phylogenetic relationships of OrM fungi isolated in the present study listed in bold. (a) relationships among *Tulasnella calospora* taxa from within the species complex with other *Tulasnella* taxa, (b) relationships among *Ceratobasidium* taxa with other members of the Ceratobasidiaceae
